# Characterization of Human Papilloma Virus in Prostate Cancer Patients Undergoing Radical Prostatectomy—A Prospective Study of 140 Patients

**DOI:** 10.3390/v15061264

**Published:** 2023-05-28

**Authors:** Tobias Nellessen, Benedikt Ebner, Nikolaos Pyrgidis, Stephan Ledderose, Alexander Kretschmer, Julian Marcon, Daniel Teupser, Doris Mayr, Valentina Faihs, Christian G. Stief, Lars E. French, Annika Herlemann, Markus Reinholz

**Affiliations:** 1 Department of Urology, Ludwig-Maximilians-University Munich, 81377 Munich, Germany; tobias.nellessen@med.uni-muenchen.de (T.N.);; 2 Department of Dermatology and Allergy, Ludwig-Maximilians-University Munich, 81377 Munich, Germany; 3 Institute of Pathology, Ludwig-Maximilians-University Munich, 81377 Munich, Germany; 4 Institute of Laboratory Medicine, Ludwig-Maximilians-University Munich, 81377 Munich, Germany; daniel.teupser@med.uni-muenchen.de; 5 Department of Dermatology and Allergy Biederstein, Technical University of Munich, 80802 Munich, Germany

**Keywords:** human papilloma virus, HPV, high-risk, low-risk, prostate cancer, radical prostatectomy

## Abstract

Introduction: The association between human papilloma virus (HPV) and the pathogenesis of prostate cancer (PCa) is still controversial. Existing studies often lack information about clinical risk factors, are limited by their retrospective design or only use a single detection method for HPV. Material and Methods: A total of 140 patients undergoing radical prostatectomy (RP) for PCa at the Department of Urology, Ludwig Maximilian University of Munich, Germany, were prospectively enrolled. Knowledge of HPV and sociodemographic parameters were assessed with questionnaires. The following methods were used for HPV detection: RP specimens were tested for HPV DNA by PCR. If HPV DNA was detected, an LCD-Array hybridization technique was used for HPV subtyping, and immunohistochemical staining for p16 was performed as a surrogate marker for HPV infection. Serological titers of HPV-16 L1 antibodies were measured using an HPV-16-specific immunoassay. Results: HPV DNA was detected in 9.3% (13/140) of RP specimens, with HPV-16 being the most predominantly detected subtype (5/13 = 39%). HPV-16 L1 antibody levels were below the limit of detection in 98% of patients (137/140). We found no significant difference between HPV PCR-positive (HPV+) and -negative (HPV-) patients in terms of HPV-16 antibody levels, history of HPV-associated diseases, level of education or marital status. Seventy-five percent of all PCa patients had never heard of HPV before. An acinar adenocarcinoma of the prostate was the most frequently detected histologic type in both HPV+ (100%) and HPV− (98%) patients (*p* = 0.86). HPV+ patients had fewer positive biopsy cores (3.5 vs. 5.8; *p* = 0.01) and a lower maximal tumor infiltration rate per core (37% vs. 57%; *p* = 0.03) compared to HPV- patients. However, when analyzing the whole prostate and the lymph nodes after RP, there were no significant differences in TNM stage, Gleason score or tumor volume between both groups. In a subgroup analysis of all high-risk HPV patients (*n* = 6), we found no significant differences in sociodemographic, clinical or histopathological parameters compared to HPV- or low-risk HPV+ patients. Conclusion: In our prospective study, we were not able to prove a clinically significant impact of HPV status on tumor characteristics in RP specimens. Most men with PCa had never heard of HPV, despite its proven causal association with other tumor entities.

## 1. Introduction

Overall, 5.2% of the global cancer burden may be attributable to human papilloma viruses (HPV), with HPV-16 or -18 being the predominant subtypes [1]. A causal relationship has been proven in a wide range of tumor entities, including cervical [2,3], oropharyngeal [4], anal [5], vulvar [6], vaginal [7] and penile [8,9] cancer. While these pathogenic links have become well established, evidence of the association between HPV and the pathogenesis of prostate cancer (PCa) remains inconclusive [10].

In a meta-analysis of 46 studies with 4919 PCa patients from Yang et al., HPV was detected in PCa tissue in 19% of cases [11]. Thirteen HPV subtypes (HPV 6, 11, 16, 18, 31, 33, 35, 39, 45, 52, 58, 59 and 68) have been described, with high-risk HPV types being predominant [11]. However, HPVs can also be detected in benign prostatic tissue [12,13] and, thus, it could be argued that the presence of HPV in PCa might be coincidental. However, in a meta-analysis, Lawson et al. included 26 case-control studies, and high-risk HPVs were identified in 325 (22.6%) of 1437 PCa patients but only in 113 (8.6%) of 1313 normal or benign prostate hyperplasia controls (*p* = 0.001) [14]. Furthermore, it has been shown that high-risk HPVs are present in prostate tissue prior to the development of HPV-positive (HPV+) PCa, suggesting an oncogenic activity for these HPV subtypes [13]. In detail, it has been suggested that HPV might modulate PCa cell behavior by affecting inflammation, angiogenesis and apoptosis mechanisms [15].

The association between HPV antibody levels and PCa is also controversial. In an analysis of men who donated serum samples in Finland, 165 PCa patients were matched with 165 control subjects. The presence of IgG antibodies against HPV-16 or -18 was associated with an increased risk of developing PCa (relative risks 2.4 and 2.6, respectively) [16]. However, in that study, the time delay between donation of the serum samples and the diagnosis of PCa was up to 24 years, constituting an immense risk of potential bias [16]. In contrast, Adami et al. reported no association between HPV-16 or -18 antibody levels and PCa risk in an analysis of 238 PCa patients and 210 population-based control subjects (OR 0.7, 95% CI 0.4–1.3) [17].

There is an urgent need to clarify the association between HPV and PCa, as PCa is the second most frequent cancer in men and the fifth leading cause of death worldwide, constituting a huge burden for global health [18]. Firstly, a causal attribution might have consequences for patient stratification. In oropharyngeal cancer, for example, HPV positivity is a strong and independent prognostic factor for better survival, as these tumors respond better to chemotherapy and radiation [19,20]. Secondly, HPVs would be the only infectious pathogen that can be prevented by vaccination [14]. It has been shown that licensed HPV vaccines are safe and highly effective and might lead to the complete prevention of HPV-associated cancers [21].

In general, existing studies on HPV and PCa often lack information about clinical risk factors, are limited by their retrospective design, or only use a single detection method for HPV [11]. Therefore, we aimed to characterize HPV in PCa patients undergoing RP in a prospective study, using tissue- and serum-based detection methods and linking these results to clinical risk factors and pathological tumor characteristics.

## 2. Material and Methods

### 2.1. Study Cohort

A total of 140 patients with prostate cancer undergoing radical prostatectomy (RP) were prospectively enrolled after informed consent at the Department of Urology, Ludwig Maximilian University of Munich, Munich, Germany, between April 2021 and August 2021. The study was approved by the university ethics committee (project number: 20–1000).

### 2.2. Questionnaire

A patient-completed paper-based questionnaire was preoperatively distributed among all participants, evaluating sociodemographic factors and HPV-associated pre-existing conditions, such as genital/skin warts and anal, penile, or oropharyngeal carcinomas. The questions of the original questionnaire are shown in Supplementary Table S2.

### 2.3. Molecular Assays and Identification of HPV by Polymerase Chain Reaction

Prior to surgery, serum was obtained from all patients and stored at −20 °C until further analysis. Serological detection of HPV-16 antibodies was performed using an HPV-16-specific, virus-like-particle (VLP)-based competitive immunoassay (PT Monitor^®^, Abviris Deutschland GmbH, Ahrensburg, Germany). The test was performed according to the manufacturer’s instructions. In short, 20 µL of patient serum was mixed with the provided HPV reagent, incubated for 5 min and pipetted onto a lateral flow test device. After 10 min, a coloured band appeared. Line intensities were read using a colourimetric reader (aLF, Qiagen Lake Constance GmbH, Stockach, Germany) and converted to concentrations using a calibration function. Each sample was measured twice, and mean values were used for analysis. The limit of detection was calculated as the limit of blank +3 standard deviations (SD). The HPV-16 antibody level was then divided into three categories: below the limit of detection (LOD), detectable low (LOD—1000 ng/mL) and detectable high (>1000 ng/mL). After surgery, histopathological analysis was performed by board-certified pathologists at the Institute of Pathology, LMU Munich. All RP specimens were reviewed to confirm tumor type and stage, and Gleason grading was performed according to the 5th World Health Organization (WHO) classification of genitourinary tumors. DNA from all RP specimen was analysed using the VisionArray^®^ HPV Chips assay (ZytoVision GmbH, Bremerhaven, Germany). Briefly, a minimum of 15ng of DNA was amplified by polymerase chain reaction (PCR), using the CE-IVD VisionArray^®^ HPV Primer Kit (ZytoVision GmbH, Bremerhaven, Germany) together with AmpliTaq Gold DNA Polymerase (Thermo Fisher Scientific, Waltham, MA, USA). VisionArray^®^ biotinylated HPV Primers target the L1 (late) region of Human HPV genomes resulting in amplicons with a length spanning 139–148 bp. PCR products were subsequently hybridized to HPV probes spotted on a glass chip covering the HPV types of: (i) low risk: 6, 11, 40, 42, 43, 44, 54, 55, 57, 61, 62, 72, 81CP8304, 83MM7, 84MM8, 90, 91; (ii) partial high risk: 26, 34, 53, 66, 67, 68a, 68b, 69, 70, 73, 82IS39, 82MM4; and (iii) high risk: 16, 18, 31, 33, 35, 39, 45, 51, 52, 56, 58, 59. As a control of functional DNA as well as the PCR, reaction primers specific for a fragment of the human HLA-DQA1 gene (amplicon length: 222 bp) were also included by the vendor. Specifically bound biotinylated HPV-specific single-strand PCR products were detected by adding streptavidin–peroxidase conjugates in the presence of tetra-methyl-benzidine (TMB), resulting in a blue precipitate. For detection and analysis, HPV types were identified by employing VisionArray^®^ Analyzer Software (ZytoVision GmbH, Bremerhaven, Germany).

### 2.4. Immunohistochemistry

For all RP specimens with a positive HPV-PCR, immunohistochemical (IHC) staining for p16 was performed on formalin-fixed, paraffin-embedded (FFPE) tissues with representative tumor sections using an antibody against p16 (clone E6H4/p16Ink4a, Ventana, ready-to-use) on a Ventana Benchmark XT autostainer (Ventana Medical Systems, Oro Valley, AZ, USA) according to the manufacturer’s protocol.

### 2.5. Statistics

Baseline variables were calculated as mean and standard deviation (SD), as frequencies with percentages or as median and interquartile range (IQR). All parameters were assessed for normality with histograms and with the Shapiro-Wilk test and the corresponding comparisons were performed using a χ2 or two-sample *t*-test. All statistical tests were performed with R (v3.6.3) and DATAtab Statistics Calculator (DATAtab e.U., Graz, Austria). A two-sided *p*-value < 0.05 was considered statistically significant.

## 3. Results

**Figure 1:**
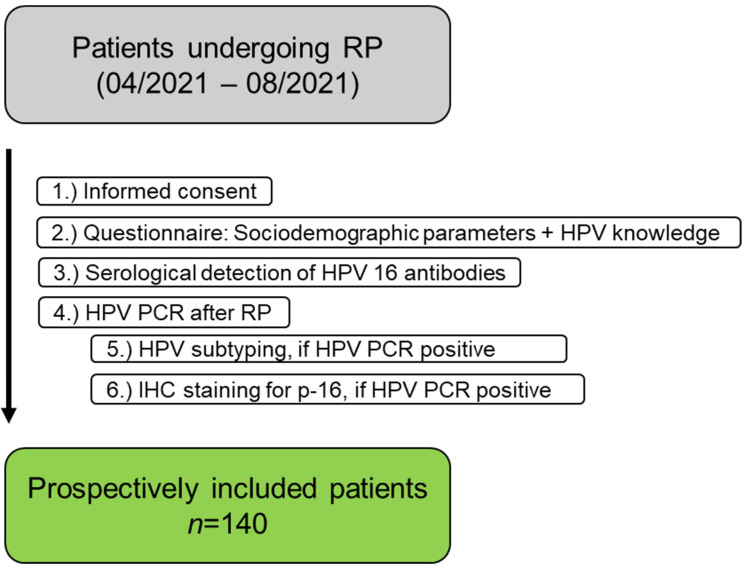
Patient Flowchart. RP: radical prostatectomy, IHC: immunohistochemical staining.

A patient flowchart is shown in Figure 1. In total, 140 PCa patients undergoing RP were prospectively included.

### 3.1. Sociodemographic Factors

Patient age, education level and marital status are presented in Table 1. Mean patient age was 66 ± 8 years. In terms of highest educational degree, 50% of patients reported lower secondary education, 8.2% reported upper secondary education and 42% reported tertiary education. In total, 8.1% of patients were single, 82% were married or in a partnership, 8.9% were divorced and 1.5% were widowed. There were no statistically significant differences in patient age, education level or marital status when comparing patients with an HPV PCR positive (HPV+) and negative (HPV-) RP specimen.

### 3.2. Patient’s Knowledge about HPV and History of HPV-Related Diseases

The patients’ knowledge about HPV and history of HPV-related diseases are shown in Table 2. Of all included PCa patients, 105 (75%) had never heard of HPV before. Patients with a tertiary education had heard of HPV more frequently than those with secondary education (odds ratio (OR) 2.53, *p* = 0.02). None of the patients were vaccinated against HPV, while 21% of patients reported that they were aware of the existence of an HPV vaccination. A total of 9.7% of the patients reported that they had suffered from skin warts before, and one patient (0.7%) reported genital warts. No other HPV-related diseases or cancers were reported. None of the patients reported the intake of systemic immunosuppressive drugs. There was one patient (0.7%) with an HIV infection treated with antiretroviral drugs. None of the patients met the criteria for hereditary prostate cancer; however, 22% of the patients (29/143) had a positive first-degree family history of PCa. There were no significant differences in terms of knowledge of HPV, history of HPV-related diseases or PCa family history between HPV+ and HPV- patients.

### 3.3. Prostate Cancer Characteristics

#### 3.3.1. Prostate Biopsy

Patient prostate biopsy results are shown in Table 3. There was no statistically significant difference in the number of obtained biopsy cores between HPV+ and HPV- patients (12.6 vs. 13.3, *p* = 0.49). HPV+ patients had fewer positive biopsy cores (3.5 vs. 5.8; *p* = 0.01) and a lower maximal tumor infiltration rate per core (37% vs. 57%; *p* = 0.03) than HPV- patients. In 90% of cases, PCa was detected in both lobes of the prostate, with no significant difference between groups (HPV+ 90% vs. HPV- 92%; *p* > 0.99). There was no significant difference between HPV+ and HPV- patients with regard to Gleason score (GS) distribution in prostate biopsies (*p* = 0.76), with GS 3 + 4 being the most frequently diagnosed GS in HPV+ and HPV- patients (31% and 32% of all cases, respectively).

#### 3.3.2. RP Specimens

Characteristics of RP specimens are presented in Table 4. The median prostate-specific antigen (PSA) at presentation before RP was 8.45 ng/mL (IQR = 5.7–14.6 ng/mL). There was no statistically significant difference between HPV+ and HPV- patients (HPV+ 7.6 ng/mL vs. HPV- 5.8 ng/mL, *p* = 0.35). RP was either performed by an open (73/140 = 52%) or robot-assisted (67/140 = 48%) approach. The median weight of the resected RP specimen was 59.8 g and median tumor volume of the RP specimen was 25%, with no statistically significant difference between HPV groups (median weight of specimen: HPV+ 62.2 g vs. HPV- 59.5 g, *p* = 0.68, median tumor volume: 22.7% vs. 24.8%, *p* = 0.70). The most common histological subtype was acinar adenocarcinoma (98% of cases). In two patients (1.4%), a small cell neuroendocrine carcinoma was found, and one patient (0.7%) was diagnosed with a ductal adenocarcinoma. Lymph nodes were removed in 116 of 140 patients (83%) and were positive in 11% (13/116). There were no statistically significant differences between HPV+ and HPV- patients in terms of histologic subtype (*p* = 0.86), GS (*p* = 0.086), local tumor stage (pT-stage, *p* = 0.15) or local nodal stage (N-status, *p* = 0.23).

#### 3.3.3. Detection of HPV in RP Specimens

**Figure 2:**
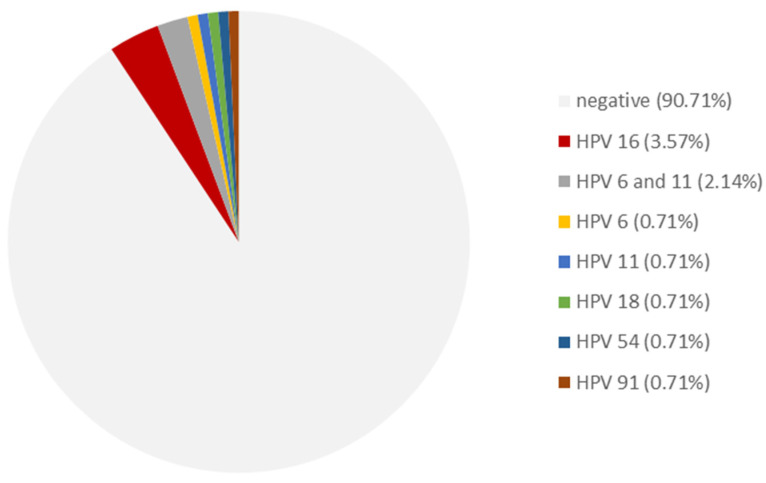
Distribution of HPV subtypes in radical prostatectomy specimen.

HPV DNA was detected in 9.3% (13/140) of RP specimens. The results of the HPV subtyping are depicted in Figure 2. HPV-16 was the most frequently detected high-risk HPV subtype (5/13 patients), followed by HPV-18 (1/13 patients). Low-risk HPV types (HPV 6, 11, 54, 91) were detected in seven patients, including three patients with a co-infection of HPV 6 and 11.

**Figure 3:**
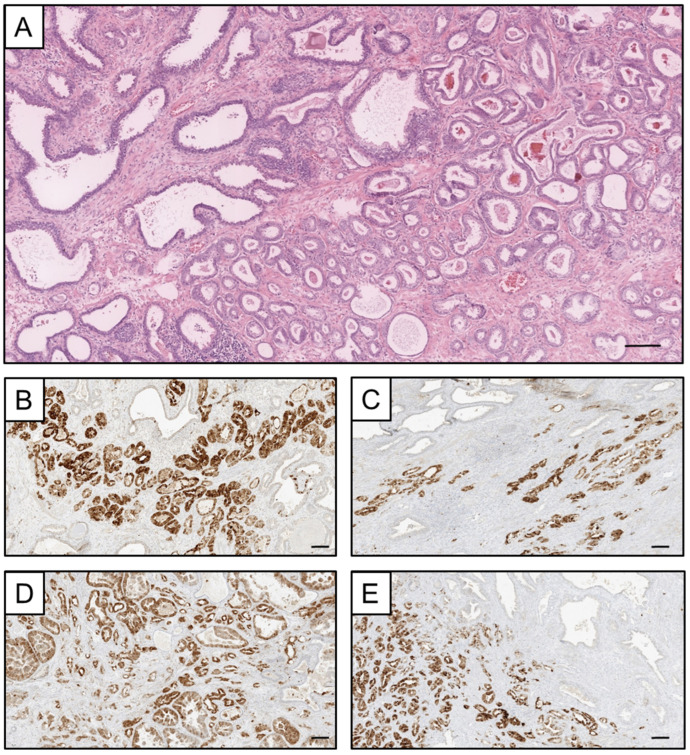
(**A**) A representative photomicrograph showing prostatic parenchyma with regular prostatic glands and intervening infiltrates of acinar prostatic carcinoma. (**B**–**E**) The atypical glands show strong immunoreactivity in p16 immunohistochemistry, while the locular glands remain negative. Scale bar 100 µm.

To discriminate between active and inactive HPV infection, IHC for P16^INK4a^ was performed on all HPV+ RP specimens (Figure 3). P16^INK4a^ was found to be overexpressed (=active HPV infection) in two of the five HPV-16-positive patients (40%) and in two of three patients with a co-infection of HPV 6 and 11 (67%). In all other HPV+ patients, IHC for P16^INK4a^ was negative.

### 3.4. HPV-16 L1 Antibody Levels

HPV-16 L1 antibody levels are presented in Table 5. They were below the limit of detection in 98% of patients (137/140). One patient was strongly seropositive with an antibody concentration of 7046 ng/mL (“detectable high”); however, HPV PCR was negative in the RP specimen of this patient. Two further patients (1.4%) had detectable antibody titres (729 ng/mL and 779 ng/mL, “detectable low”). In one of them, HPV 6 DNA was detected in the RP specimen. None of the patients with detectable HPV-16 L1 antibodies reported other HPV-related diseases or cancers in their medical history.

All five HPV-16+ patients had HPV-16 L1 antibody levels below the limit of detection. There was no statistically significant difference between HPV+ and HPV- patients regarding HPV-16 L1 antibody level (*p* = 0.13). We further performed an analysis that only included high-risk HPV+ patients; but again, there was no statistically significant difference in HPV-16 L1 antibody levels between high-risk HPV+ and all other patients (HPV- and low-risk HPV+; *p* = 0.93; Supplementary Table S1).

## 4. Discussion

PCa is the second most frequent cancer in men and the fifth leading cause of death worldwide [18]. Despite the proven causal association between HPV and several other tumor entities [11], there is still no consensus about a possible pathogenic link between HPVs and PCa, as existing studies report controversial results [11].

HPVs would be the only infectious pathogen in PCa that can be prevented by vaccination [14]. Furthermore, HPVs may act as response markers for chemotherapy and radiation in specific tumors [19,20] and thus might play an important role in patient stratification. Existing studies often lack information about clinical risk factors, are limited by their retrospective design, or only use a single detection method for HPV. We prospectively characterized HPV in PCa patients undergoing RP, using tissue- as well as serum-based detection methods and linked these results to clinical risk factors and pathological tumor characteristics.

Our findings indicate that there is no clinically significant impact of HPV status on tumor characteristics in RP specimens. HPV+ patients had fewer positive biopsy cores and a lower maximal tumor infiltration rate per core, but there was no significant difference between HPV+ and HPV- patients in terms of histologic subtype, GS, local tumor stage or local nodal stage when analyzing the whole prostate (and lymph nodes) after RP. In our study, HPV DNA was detected in 9.3%, which is lower in comparison to a meta-analysis from Yang et al. that included 46 studies with 4919 PCa patients and reported a 19% prevalence of HPV infection [11].

HPV-16 L1 antibody levels were below the limit of detection in the vast majority of PCa patients (98%) in our study, and there was no association between detectable HPV-16 L1 antibody levels and HPV PCR status. A recent systematic review listed 15 serology studies of HPV in prostate cancer [14]. The percentage of patients with detectable antibodies to HPV varied between the used methods and ranged from 4% to 68% in prostate cancer patients as well as in the control groups (4–68%) [14]. In our study, we only tested for HPV-16, as this was the predominant subtype in the analysis of the RP specimens.

We found no significant differences in sociodemographic factors such as patient age, education level or marital status when comparing HPV+ and HPV- patients. Furthermore, there were no differences in terms of knowledge of HPV, history of HPV-related diseases or PCa family history between HPV+ and HVP- patients. Most PCa patients had never heard of HPV before, despite its proven causal association with several tumor entities [14]. Patients with a tertiary education had heard of HPV more frequently than those with only secondary education, proving the importance of educational programs for HPV awareness, which has been discussed before [22].

Overall, 5.2% of the global cancer burden might be attributable to HPVs [1]. It has been pointed out before that effective communication of the safety and efficacy of HPV vaccination is critical to promote global implementation and fully realize the potential for the prevention of HPV-related cancers [21]. Our study clearly demonstrates, in a group of elderly German men, that basic knowledge about HPV often does not exist. Men serve a key role in spreading HPV [23]. However, a recent systematic literature review on HPV knowledge reported that the percentage of female adolescents knowing about HPV is consistently higher than that of boys (16.4–92.8% vs. 8.1–51.3%) [22]. A clear association between HPV and PCa could reinforce the importance of effective and gender-neutral HPV vaccination campaigns [10].

To understand the complex interplay between viral and host factors that might be the reason for the incongruity of evidence and to find the best approach for prevention, early diagnosis and treatment, many efforts are still necessary [24]. It has been shown that E7, one of the major HPV oncoproteins, might be one of the fundamental contributors that induces carcinogenesis [25]. E7 inactivates retinoblastoma protein by phosphorylation. This leads to an increase in free eukaryotic transcription factor E2F, followed by an increase in p16 [26]. In cervical cancer, immunohistochemical evaluation of E7 oncoprotein staining has been reported to be feasible [27] and might be a more adequate surrogate marker for HPV, as p16 is not exclusively increased by E7 oncoprotein in carcinogenesis. Further studies exploring the association between HPV and PCa on a molecular level are needed.

## 5. Limitations

It should be noted that despite our extensive approach with prospectively collected data, there are several major limitations to the present study. First and foremost, although all patients were prospectively enrolled, we used a single-center setting without a control group. Second, only including patients who underwent RP in our department may have led to a selection bias, especially in low-risk PCa patients, who often choose active surveillance. Third, we only tested for HPV-16 L1 antibody levels, not for antibody titers of other HPV types. Fourth, the absence of HPV in RP specimens does not rule out HPV infections in the past. Fifth, expression levels of E7 might be a more specific surrogate marker for HPV than p16 expression levels.

## 6. Conclusions

We were not able to prove a clinically significant impact of HPV status on tumor characteristics in RP specimens. Furthermore, HPV-16 L1 antibody levels were below the limit of detection in the vast majority of PCa patients (98%) in our study, and there was no association between detectable HPV-16 L1 antibody levels and HPV PCR status. Most men with PCa have never heard of HPV, despite its proven causal association with other tumor entities. Continued efforts should be made to raise awareness for HPV-related diseases and vaccination programs.

## Figures and Tables

**Table 1:** Age, marital status and education level. HPV-: HPV PCR negative, HPV+: HPV PCR positive, RP: radical prostatectomy.

	Overall Cohort *n* = 140	HPV- RP Specimen, *n* = 127	HPV+ RP Specimen, *n* = 13	*p*-Value
Age (years)	66.4 ± 8.0	66.6 ± 8.2	64.4 ± 6.5	0.26
Marital status				0.70
Divorced	12 (8.9%)	11 (8.9%)	1 (8.3%)	
Married or relationship	110 (81%)	101 (82%)	9 (75%)	
Single	11 (8.1%)	9 (7.3%)	2 (17%)	
Widowed	2 (1.5%)	2 (1.6%)	0 (0%)	
Highest level of education				0.072
Lower secondary	67 (50%)	63 (52%)	4 (33%)	
Upper secondary	11 (8.2%)	8 (6.6%)	3 (25%)	
Tertiary	56 (42%)	51 (42%)	5 (42%)	

**Table 2:** Patient’s knowledge about HPV and history of HPV-related diseases. HPV-: HPV PCR-negative, HPV+: HPV PCR-positive, PCa: prostate cancer, RP: radical prostatectomy.

	Overall Cohort, *n* = 140	HPV- RP Specimen, *n* = 127	HPV+ RP Specimen, *n* = 13	*p*-Value
Knowledge of existence of HPV	35 (25%)	33 (26%)	2 (17%)	0.71
Knowledge of vaccine against HPV	29 (21%)	27 (21%)	2 (17%)	0.99
Vaccinated against HPV				
Yes	0 (0%)	0 (0%)	0 (0%)	
HPV-related diseases	13 (9.7%)	11 (9.0%)	2 (17%)	0.73
Immunosuppression	1 (HIV, 0.7%)	1 (HIV, 0.8%)	0 (0%)	>0.99
Positive family history of PCa	29 (22%)	27 (22%)	2 (17%)	0.94

**Table 3:** Histopathological characteristics after prostate biopsy. HPV-: HPV PCR negative, HPV+: HPV PCR positive, bilateral: both prostate lobes, unilateral: one prostate lobe, RP: radical prostatectomy.

	Overall Cohort, *n* = 140	HPV- RP Specimen, *n* = 127	HPV+ RP Specimen, *n* = 13	*p*-Value
Gleason score after biopsy				0.76
6	28 (22%)	24 (21%)	4 (31%)	
3 + 4	40 (31%)	36 (32%)	4 (31%)	
4 + 3	16 (13%)	14 (12%)	2 (15%)	
8	26 (20%)	23 (20%)	3 (23%)	
9	16 (13%)	16 (14%)	0 (0%)	
10	1 (0.8%)	1 (0.9%)	0 (0%)	
No. of positive biopsy cores [*n*]	5.6 ± 3.5	5.8 ± 3.6	3.5 ± 2.6	0.010
No. of total biopsy cores [*n*]	12.7 ± 3.5	12.6 ± 3.5	13.3 ± 3.4	0.49
Maximal tumor infiltration rate per core [%]	54.5 ± 28.9	56.6 ± 28.7	36.8 ± 25.5	0.032
Biopsy: uni/bilateral tumor				>0.99
Bilateral	123 (90%)	111 (90%)	12 (92%)	
Unilateral	13 (9.6%)	12 (9.8%)	1 (7.7%)	

**Table 4:** Histopathological characteristics after radical prostatectomy. HPV-: HPV PCR negative, HPV+: HPV PCR positive, PCa: prostate cancer, RP: radical prostatectomy.

	Overall Cohort, *n* = 140	HPV- RP Specimen, *n* = 127	HPV+ RP Specimen, *n* = 13	*p*-Value
PSA at RP	Median 8.4 (IQR 5.7–14.6)	Median 8.5 (IQR 5.8–16.3)	Median 7.6 (IQR 5.0–12.0)	0.35
Weight of RP specimen [g]	59.8 ± 23.3	59.5 ± 24.2	62.2 ± 11.8	0.68
Gleason score after RP				0.086
6	21 (15%)	16 (13%)	5 (38%)	
3 + 4	59 (43%)	53 (43%)	6 (46%)	
4 + 3	25 (18%)	25 (20%)	0 (0%)	
8	12 (8.8%)	11 (8.9%)	1 (7.7%)	
9	20 (15%)	19 (15%)	1 (7.7%)	
Tumor volume of RP specimen [%]	24.6 ± 18.2	24.8 ± 18.3	22.7 ± 17.7	0.70
Histologic subtype				0.86
Acinar adenocarcinoma	135 (98%)	123 (98%)	12 (100%)	
Ductal adenocarcinoma	1 (0.7%)	1 (0.8%)	0 (0%)	
Small cell neuroendocrine	2 (1.4%)	2 (1.6%)	0 (0%)	
Pathologic T-stage				0.15
pT2a	10 (7.1%)	10 (7.9%)	0 (0%)	
pT2b	2 (1.4%)	1 (0.8%)	1 (7.7%)	
pT2c	76 (54%)	66 (52%)	10 (77%)	
pT3a	24 (17%)	23 (18%)	1 (7.7%)	
pT3b	26 (19%)	25 (20%)	1 (7.7%)	
pT4	2 (1.4%)	2 (1.6%)	0 (0%)	
Pathologic N-stage				0.23
pN0	103 (74%)	94 (74%)	9 (69%)	
pN1	13 (9.3%)	13 (10%)	0 (0%)	
pNx	24 (17%)	20 (16%)	4 (31%)	

**Table 5:** HPV-16 L1 antibody levels. HPV-: HPV PCR negative, HPV+: HPV PCR positive, RP: radical prostatectomy.

	Overall Cohort, *n* = 140	HPV- RP Specimen, *n* = 127	HPV+ RP Specimen, *n* = 13	*p*-Value
Level of HPV-16 L1 antibodies (category)				0.13
Below detection limit	137 (98%)	125 (98%)	12 (92%)	
Detectable low	2 (1.4%)	1 (0.8%)	1 (7.7%)	
Detectable high	1 (0.7%)	1 (0.8%)	0 (0%)	

## Data Availability

The data presented in this study are available on request from the corresponding author. The data are not fully publicly available yet due to further ongoing studies, that are not published yet.

## Supplementary Materials

The following supporting information can be downloaded at: https://www.mdpi.com/article/10.3390/v15061264/s1, Table S1: Baseline characteristics and univariate analysis of patients with detection of high risk HPV in RP specimen vs. negative HPV PCR in RP specimen; Table S2: Patient questionnaire.
