# Supplementary material for: Characterization of Human Papilloma Virus in Prostate Cancer Patients Undergoing Radical Prostatectomy—A Prospective Study of 140 Patients
Source: Viruses. 2023 May 28;15(6):1264. doi: 10.3390/v15061264 (PMC10302226; doi:10.3390/v15061264)
Supplement: Supplementary file 1 [file viruses-15-01264-s001.zip › viruses-2408185-supplementary.pdf]

Supplementary data

Characterization of Human Papilloma Virus in Prostate Cancer Patients Undergoing Radical Prostatectomy—A Prospective Study of 140 Patients

Supplementary Table S1: Baseline characteristics and univariate analysis of patients with detection of high risk HPV in RP specimen vs. negative HPV PCR in RP specimen. HPV-: HPV PCR negative, HPV+: HPV PCR positive, RP: radical prostatectomy, bilateral: both prostate lobes, unilateral: one prostate lobe.

	**Overall cohort**, n = 140	**HPV- or low-risk HPV+ in RP specimen**, n = 134	**High-risk HPV+ in RP specimen**, n = 6	**p-value**
**Age**	66.4 ± 8.0	66.6 ± 8.1	62.8 ± 5.9	0.19
**Marital status**				0.13
Divorced	12 (8.9%)	12 (9.3%)	0 (0%)	
Married or relationship	110 (81%)	106 (82%)	4 (67%)	
Single	11 (8.1%)	9 (7.0%)	2 (33%)	
Widowed	2 (1.5%)	2 (1.6%)	0 (0%)	
**Highest level of education**				0.071
Lower secondary	67 (50%)	65 (51%)	2 (33%)	
Upper secondary	11 (8.2%)	9 (7.0%)	2 (33%)	
Tertiary	56 (42%)	54 (42%)	2 (33%)	
**Knowledge of existence of HPV**	35 (25%)	34 (26%)	1 (17%)	0.98
**Knowledge of vaccine against HPV**	29 (21%)	28 (21%)	1 (17%)	>0.99
**Vaccinated against HPV**				
Yes	0 (0%)	0 (0%)	0 (0%)	
**HPV-related diseases**	13 (9.7%)	12 (9.4%)	1 (17%)	>0.99
**Immunosuppression**	1 (HIV, 0.7%)	1 (HIV, 0.8%)	0 (0%)	>0.99
**Positive family history of PCa**	29 (22%)	29 (23%)	0 (0%)	0.42
**Gleason score after biopsy**				0.82
6	28 (22%)	26 (21%)	2 (33%)	
3+4	40 (31%)	39 (32%)	1 (17%)	

	Overall cohort, n = 140	HPV- or low-risk HPV+ in RP specimen, n = 134	High-risk HPV+ in RP specimen, n = 6	p-value
4+3	16 (13%)	15 (12%)	1 (17%)	
8	26 (20%)	24 (20%)	2 (33%)	
9	16 (13%)	16 (13%)	0 (0%)	
10	1 (0.8%)	1 (0.8%)	0 (0%)	
**No. of positive biopsy cores [n]**	5.6 ± 3.5	5.6 ± 3.6	4.5 ± 3.3	0.45
**No. of total biopsy cores [n]**	12.7 ± 3.5	12.7 ± 3.5	11.8 ± 3.4	0.56
**Maximal tumor infiltration rate per core [%]**	54.5 ± 28.9	54.8 ± 29.2	50.0 ± 25.5	0.68
**Biopsy: uni/bilateral tumor**				>0.99
Bilateral	123 (90%)	118 (91%)	5 (83%)	
Unilateral	13 (9.6%)	12 (9.2%)	1 (17%)	
**PSA at RP**	Median 8.4 (IQR 5.7-14.6)	Median 8.4 (IQR 5.7-14.9)	Median 10.1 (IQR 5.8-13.1)	0.95
**Weight of RP specimen [g]**	59.8 ± 23.3	59.6 ± 24.0	61.2 ± 13.4	0.84
**Gleason score after RP**				0.11
6	21 (15%)	18 (14%)	3 (50%)	
3+4	59 (43%)	57 (44%)	2 (33%)	
4+3	25 (18%)	25 (19%)	0 (0%)	
8	12 (8.8%)	11 (8.4%)	1 (17%)	
9	20 (15%)	20 (15%)	0 (0%)	
**Tumor volume of RP specimen [%]**	24.6 ± 18.2	24.4 ± 18.3	27.5 ± 14.7	0.64
**Histologic subtype**				0.93
Acinar adenocarcinoma	135 (98%)	129 (98%)	6 (100%)	
Ductal adenocarcinoma	1 (0.7%)	1 (0.8%)	0 (0%)	
Small cell neuroendocrine	2 (1.4%)	2 (1.5%)	0 (0%)	
**Pathologic N-stage**				0.45
pN0	103 (74%)	99 (74%)	4 (67%)	
pN1	13 (9.3%)	13 (9.7%)	0 (0%)	
pNx	24 (17%)	22 (16%)	2 (33%)	
**Pathologic T-stage**				**0.034**
pT2a	10 (7.1%)	10 (7.5%)	0 (0%)	
pT2b	2 (1.4%)	1 (0.7%)	1 (17%)	
pT2c	76 (54%)	72 (54%)	4 (67%)	

	**Overall cohort**, n = 140	**HPV- or low-risk HPV+ in RP specimen**, n = 134	**High-risk HPV+ in RP specimen**, n = 6	**p-value**
pT3a	24 (17%)	23 (17%)	1 (17%)	
pT3b	26 (19%)	26 (19%)	0 (0%)	
pT4	2 (1.4%)	2 (1.5%)	0 (0%)	
**IHC p-16 positive**	4 (31%)	2 (29%)	2 (33%)	>0.99
**Level of HPV-16 L1 antibodies antibodies (category)**				0.93
Below detection limit	137 (98%)	131 (98%)	6 (100%)	
Detectable high	1 (0.7%)	1 (0.7%)	0 (0%)	
Detectable low	2 (1.4%)	2 (1.5%)	0 (0%)	

Supplementary Table S2: Patient *questionnaire*

1. Please provide your age:

____________Years

2. Marital status

(1)☐Single (2)☐Married/partnership
(3)☐Divorced (4)☐Widowed

3. What is your highest level of education?

(1)☐No degree (2)☐ Elementary school certificate
(3)☐Lower secondary school certificate (4)☐ Upper secondary school certificate
(5)☐Tertiary school certificate

4. What is your current health insurance?

(1)☐ State insurance (2)☐Private insurance (3)☐No insurance

5. When did the core biopsy of your prostate first show cancer?
Date: __________

6. Have you ever heard of the Human Papillomavirus (HPV)?
(1)☐Yes (2)☐No

7. Are you aware that there is a vaccination against HPV?
(1)☐Yes (2)☐No

8. Are you vaccinated against HPV (Cervarix, Gardasil, Gardasil9)?
(1)☐Yes (2)☐No

9. Do you suffer or have you suffered from other HPV-associated diseases? (Examples see question 10)
(1)☐Yes (2)☐No

10. If yes, which HPV-associated diseases do/did you suffer from?

(1)☐Genital warts
(2)☐Skin warts
(3)☐Anal carcinoma
(4)☐Penile carcinoma
(5)☐Carcinomas of the mouth, throat and larynx (oropharyngeal carcinomas)
(6)☐Other: ____________

11. Do you suffer from any diseases affecting the immune system or do you take any immunosuppressing drugs?

(1)☐Yes (2)☐No
If yes, please provide details of the illness/medication: _______________________

12. Are there any other cases of prostate cancer in your family? If so, what is the family relationship and how old was the person at the time of diagnosis (e.g. "father, 55 years old")?
____
